# Parasagittal Approach of Epidural Steroid Injection as a Treatment for Chronic Low Back Pain: A Systematic Review and Meta-Analysis

**DOI:** 10.3389/fpain.2021.676730

**Published:** 2021-06-30

**Authors:** Nebojsa Nick Knezevic, Stephania Paredes, Santiago Cantillo, Albara Hamid, Kenneth D. Candido

**Affiliations:** ^1^Department of Anesthesiology, Advocate Illinois Masonic Medical Center, Chicago, IL, United States; ^2^Department of Anesthesiology, University of Illinois, Chicago, IL, United States; ^3^Department of Surgery, University of Illinois, Chicago, IL, United States

**Keywords:** epidural steroid injection, parasagittal interlaminar, midline interlaminar, transforaminal, chronic low back pain

## Abstract

**Background:** Epidural steroid injection (ESI) has proven benefits in controlling chronic low back pain and can be performed *via* the midline interlaminar (MIL) or transforaminal (TF) approach. A modified interlaminar approach, the parasagittal interlaminar (PIL), has surfaced as a more reliable, safe, and suitable approach to minimize complications related to MIL and TF.

**Objective:** To conduct a systematic review and meta-analysis to assess and compare the effectiveness and safety of PIL with both MIL and TF approaches in adult patients with a history of chronic low back pain.

**Methods:** A literature search was conducted using major electronic databases, such as PubMed, EMBASE, and Cochrane. Selected studies included patients with unilateral radicular symptoms, secondary to lumbar intervertebral disc hernias or degenerative lumbar disc disease, that, additionally, received ESIs *via* PIL or either MIL or TF under fluoroscopic guidance. Randomized and observational studies with pain relief score and/or functional disability assessment and at least a 2-week follow-up were included.

**Results:** The search led to the initial identification of 174 studies. Following the screening, eight studies were included in the qualitative analysis and seven randomized controlled trials (RCTs) were included in the statistical analysis. PIL showed statistically significantly more pain relief and functional improvement than MIL at 1-, 3-, and 6-month post-procedure. Compared to TF, PIL showed statistically significantly more pain relief at 3- and 6-month after the procedure. Additionally, PIL showed benefits in terms of lower mean fluoroscopy time, less radiation exposure, zero adverse events in all the included studies, no cases of intravascular spread compared with the TF approach, and a higher anterior epidural spread (AES) of PIL compared with TF.

**Conclusions:** Our systematic review and meta-analysis suggest that the PIL approach is an effective and safe alternative to the MIL and TF approaches in patients presenting with chronic low back pain when epidural injections are indicated, demonstrating a higher level of pain relief and a stronger improvement in functionality post-procedure.

## Introduction

Low back pain is the most common type of chronic pain and the leading cause of disability in the United States (1, 2). Its prevalence and healthcare expenditures are exponentially increasing. A recent investigation reported a total cost of $1.8 billion from October 2018 to March 2019 (3). At the same time, the treatment options have changed and alternative approaches are being studied for these patients. Currently, minimally invasive procedures are preferred for this population (4–6).

Epidural injections are one of the cornerstone procedures used to treat low back pain, especially when the cause is related to a herniated disc or a spinal stenosis (7). The first epidural injection was performed back in 1901 in Paris by Jean Sicard (1872–1929) and Fernand Cathelin (1873–1945) using a ureteral catheter, and steroids were added to the injections in the early 1950s (8). Steroids in epidural injections act primarily through cytokine suppression. Corticosteroids have both direct and indirect roles, decreasing the production and release of multiple pro-inflammatory cytokines, and inhibiting phospholipase A2 and the arachidonic acid pathway (4, 9, 10). Steroids also inhibit the transcription of inflammatory genes, such as nuclear factor kappa-light-chain-enhancer of activated B cells (NF-kB), and, upon binding to glucocorticoid-responsive elements (GREs), can increase the production of anti-inflammatory genes (11).

The use of these injections has historically been controversial, and multiple studies have shown variable results, both supporting and discouraging this management (12–15). However, as multiple studies associate steroids with higher pain relief scores compared with local anesthetics alone, there is an increasing interest in assessing this topic and promoting their use (7, 11). To determine the real effect of epidural steroid injections (ESIs), researchers have focused not only on the types of drugs used but also on the other factors that affect their efficacy, such as the different existing injection approaches and the performance technique (4).

**Figure 1:**
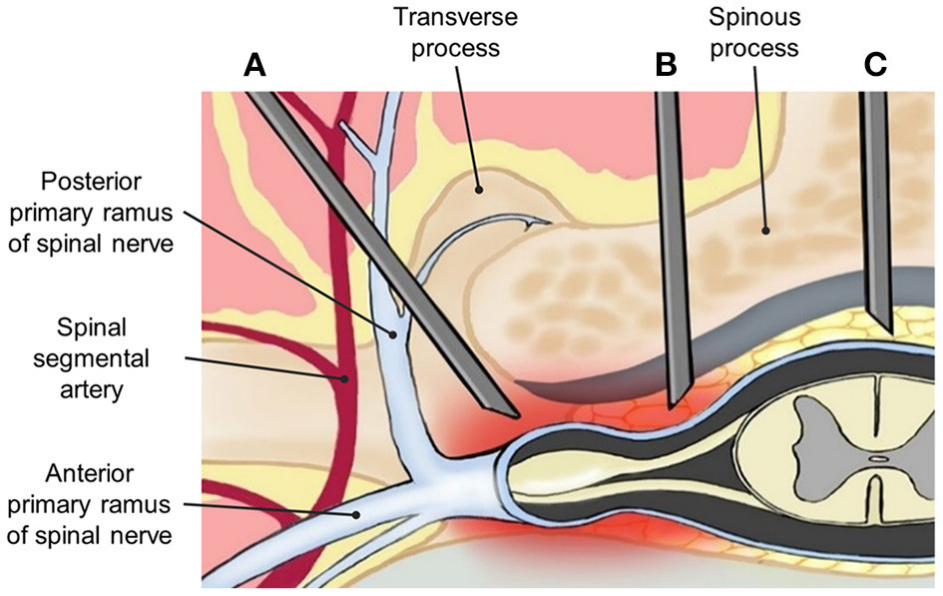
Anatomical demonstration of needle placement in **(A)** transforaminal (TF), **(B)** parasagittal interlaminar (PIL), and **(C)** midline interlaminar (MIL) epidural injections.

Lumbar epidural steroid injection (LESI) can be performed *via* the midline interlaminar (MIL) (Figure 1C) or transforaminal (TF) approach (Figure 1A), both of which have proven benefits in controlling chronic low back pain (16). A caudal approach may also be considered in selective cases. TF approach is sometimes preferred by pain physicians. Among its benefits, this approach allows a larger drug concentration to reach the anterior epidural space (AES), where the pro-inflammatory substances, including substance P, are commonly found (17, 18). However, TF has been associated with complications such as intravascular injection and nerve injury, which may lead to paresthesia, paraplegia, permanent paralysis, and even death (19–21). Therefore, multiple efforts are being made to find an effective and safer approach to deliver medications into the epidural space.

The interlaminar (IL) approach is technically less challenging compared with the TF approach and has demonstrated a lower incidence of adverse events. However, due to the anatomical location of the inserted needle, it may deliver a lower amount of drug into the AES. Thus, it is believed that clinical improvement could be limited (4, 9, 22). A modified IL approach is called the parasagittal interlaminar (PIL) approach, whereby the needle is located in the lateral part of the interlaminar space, and more directed to the nerve roots (Figure 1B). PIL has shown an 89–100% ventral spread of contrast dye in some studies, compared with just a 31.7% of the conventional IL approach (23, 24). Additionally, pain relief measured by visual analog scale (VAS) changes and functional improvement in disability measure by Oswestry Disability Index (ODI) has been superior to the midline IL ESI approach in multiple studies (22).

Demonstrating the effectiveness and superiority of each approach is challenging and depends on factors such as the disease, the expertise of the pain physician, and the patient themself. Studies have shown controversial results when comparing these three approaches. We conducted a systematic review and meta-analysis to assess and compare the effectiveness and safety of PIL compared with both MIL and TF approaches in adult patients with a history of chronic low back pain.

## Methods

### Protocol and Registration

We conducted this systematic review following the recommended data of the methodological and reporting quality of systematic reviews, preferred reporting items for systematic reviews and meta-analysis (PRISMA) (25). This systematic review and meta-analysis assessed the effectiveness and safety of the PIL epidural injection approach compared with MIL and TF approaches.

### Eligibility Criteria

#### Types of Studies

We included eight studies from 2006 to 2020. All the included studies were prospective randomized clinical trials. A detailed description of the selected studies is presented in Table 1.

**Table 1:** Characteristics of the included studies in this meta-analysis.

**Study, country, (Reference)**	**Year**	**Study design**	**Primary outcome**	**Secondary outcomes**	**Following period**	**Type of epidural injection** **approach (*****N*** **of patients)**			**Main findings**
						**MIL**	**TF**	**PIL**	
Candido et al., US (23)	2008	Prospective randomized observational study.	Contrast spread of the epidural injection.	Pain improvement (VAS score) and Fluoroscopy time	2 weeks 1month 3 months 6 months	–	28	28	All patients in PIL group (28/28) and 75% (21/28) in TF group presents anterior epidural spread. Ninety-seven percentage in PIL group and 64% in TF presented both anterior and posterior spread; and 0% in PIL and 25% in TF group had just posterior spread. Mean fluoroscopy time was statistical significant less in PIL group. VAS pain score was not significant different among groups.
Candido et al., US (26)	2013	Prospective randomized single-blinded study	Presence and severity of pressure paresthesia in the same side (concordant) or other side (discordant).	Disability index (ODI score), pain scores (NRS score) at rest and during movement, use of pain medications, and any side effect.	1, 7, 14, 21 days 1 month 2 months 3 months 6 months 1 year	50	–	50	PIL group described significantly higher rates of concordant moderate-to-severe pressure paresthesia. PIL group approach had statistically and clinically longer pain relief than MIL approach. PIL group presented slightly better quality of life scores and they also used less pain medications than MIL approach.
Ghai et al., India (22)	2013	RCT, double blind.	Pain assessment (VAS score). Relief was determined by >50% of improvement in pain score.	Disability and impairment assessment using (MODQ score) and possible neurologic complications.	15 days 1 month 2 months 3 months 6 months	18	–	19	With the PIL approach, the number of patients achieving effective pain relief was significantly higher compared with the MIL group. VAS and ODQ scores were significantly lower in the PIL group compared with the MIL group at different time intervals except baseline. No complications were identified.
Ghai et al., India (27)	2014	Prospective, randomized, double blind.	Assessed for pain (VAS score). Effectiveness was defined as a pain relief ≥ 50% reduction from baseline (VAS).	Assessed for functional impairment (MODQ) and possible neurologic complications.	2 weeks 1 month 2 moths 3 months 6 months 9 months 1 year	–	30	32	The proportion of relief was within equivalence width in the 2 groups. Between-group analysis revealed that VAS and MODQ scores were comparable in the 2 groups at all time intervals Intravascular spread was noted in three patients in the TF group.
Hashemi et al., Iran (28)	2015	Prospective cohort RCT, double blind	Effective pain relief (NRS <3).	Improvement in disability (measured by ODI <20%). Incidence of Adverse effects.	4 weeks	–	28	28	Mean NRS score and disability score were not significantly different in PIL group, compared to TF, at 4 weeks. No adverse effects were identified.
Hong et al., Korea (16)	2017	Prospective, single center, randomizedand blinded study	Relief in pain (NRS) and disability level (ODI)	Presence of concordant paresthesia, anterior epidural spread, total procedure time, and exposed radiation dose	2 weeks	–	31	41	Both the PIL and TF approach produced similar clinically significant improvements in pain and level of disability. The PIL group showed a significantly lower radiation dose and shorter procedure time.
Kaur et al., India (29)	2017	Prospective RCT, double blind	Pain improvement (VAS score)	Improvement in QOL (Quebec and depression score)	1 week 1 month 3 months	20	20	20	Statistical significant difference in change of VAS, Quebec, and depression scores from baseline. No statistical significant change in VAS score, Quebec score, and depression score among groups.
Makkar et al., India (30)	2019	Randomized double-blind trial	Effectiveness of intervention (50% reduction in VAS score at 6 months)	Change in VAS and MODQ score, contrast medium spread, Number of ESI required.	2 weeks 4 weeks 3 months 6 months	21	20	20	Significant improvement of pain with PIL and TF approaches compared with MIL. Les VAS score and better MODQ score at 6 months. Better spread of steroids with PIL and TF compared with MIL.

*N, number; RCT, Randomized controlled trial; MIL, Midline interlaminar; TF, Transforaminal; PIL, Parasagittal interlaminar; VAS, Visual analog scale; MODQ, Modified Oswestry disability questionnaire; ODI, Oswestry disability index; NRS, numeric rating scale; QOL, quality of life; ESI, Epidural steroids injection*.

#### Types of Participants

All patients in the review had unilateral radicular pain secondary to intervertebral disc herniation or degenerative lumbar disc disease. Additionally, they received treatment with ESIs, either by the PIL approach, MIL approach, or TF approach.

#### Types of Interventions

Epidural steroid injection is performed *via* the PIL approach, MIL approach, or TF approach. Fluoroscopy was used as a guidance method in all the included studies. Different types of steroids were administrated as analgesics and anti-inflammatory drugs. The patients were evaluated from a minimum of 2 weeks to 6 months after the procedure.

#### Types of Outcome Measures

The primary outcomes of interest were the degree of change in pain, measured with the VAS or numeric rating scale (NRS), and the change of functionality measured with ODI. Secondary outcomes included the rate of complications between approaches and their durations.

### Information Sources

The literature search was performed from 2006 to 2020, looking for specific studies that met our inclusion criteria. The search was performed on a variety of major electronic databases including PubMed (MEDLINE), EMBASE, and Cochrane. Previous systematic reviews were evaluated, and cross-references were used to find clinical data about our point of interest.

### Search Methodology

The search was conducted on the aforementioned databases using the following terms and keywords: “Lower back pain,” “Lumbar disc herniation,” “Degenerative disc disease,” “Chronic lumbar pain,” “Back pain with radiation,” “Unilateral radiculopathy,” “Spinal stenosis,” “Steroid injection,” “Epidural injection,” “Transforaminal,” “Interlaminar,” and “Parasagittal.” It was complemented by combining the MeSH terms “Low back pain,” “Drug Therapy,” and “Steroids.” Reviewed data were later reported using the standard recommendations provided by the PRISMA group. Articles published by December 2019 were classified to include clinical trials and observational studies if available in English. Articles were manually screened to include references unidentified by the initial search.

### Study Selection

Selected articles were filtered according to the following inclusion criteria: (1) Included patients had unilateral radicular symptoms secondary to lumbar intervertebral disc herniation or degenerative lumbar disc disease, (2) fluoroscopy was consistently used as guidance method for the procedures, (3) PIL was performed and compared with either MIL or TF approach, (4) study design included published randomized controlled trials (RCTs) or observational (prospective or retrospective) studies, (5) outcome variables included VAS, NRS, or ODI, and (6) patients were followed for at least 2 weeks. Subsequently, eligible articles were evaluated based on the exclusion criteria: (1) case reports, review articles, letters to the editor, and abstracts; (2) articles that included patients with possible or confirmed spinal stenosis; (3) articles where the outcomes of interest were not reported, and (4) non-English study articles.

### Data Collection Process

Two investigators (SP and SC) independently assessed the full text when the inclusion criteria were fulfilled, and the study data and variables were considered probably relevant. Some authors of the selected articles with important data were contacted *via* email to collect additional data not presented in the published version of the study. When communication with authors was not successful, data were recollected by graphics and converted from the available data, such as median and interquartile range to mean and SD, through formulas presented in the Cochrane Handbook for Systematic Reviews of Interventions, version 5.1.0 (31). Discrepant opinions were discussed by investigators until a consensus was reached.

### Data Items

The names of the authors, year of publication, type of study, type of procedural approach and sample size in each arm of the trial/observational study, randomization technique, blinded technique, length of follow-up, and primary and secondary outcomes of each study were extracted, as well as possible complications and important findings possibly related to the approach type reported in the study.

### Risk of Bias in Individual Studies

**Figure 2:**
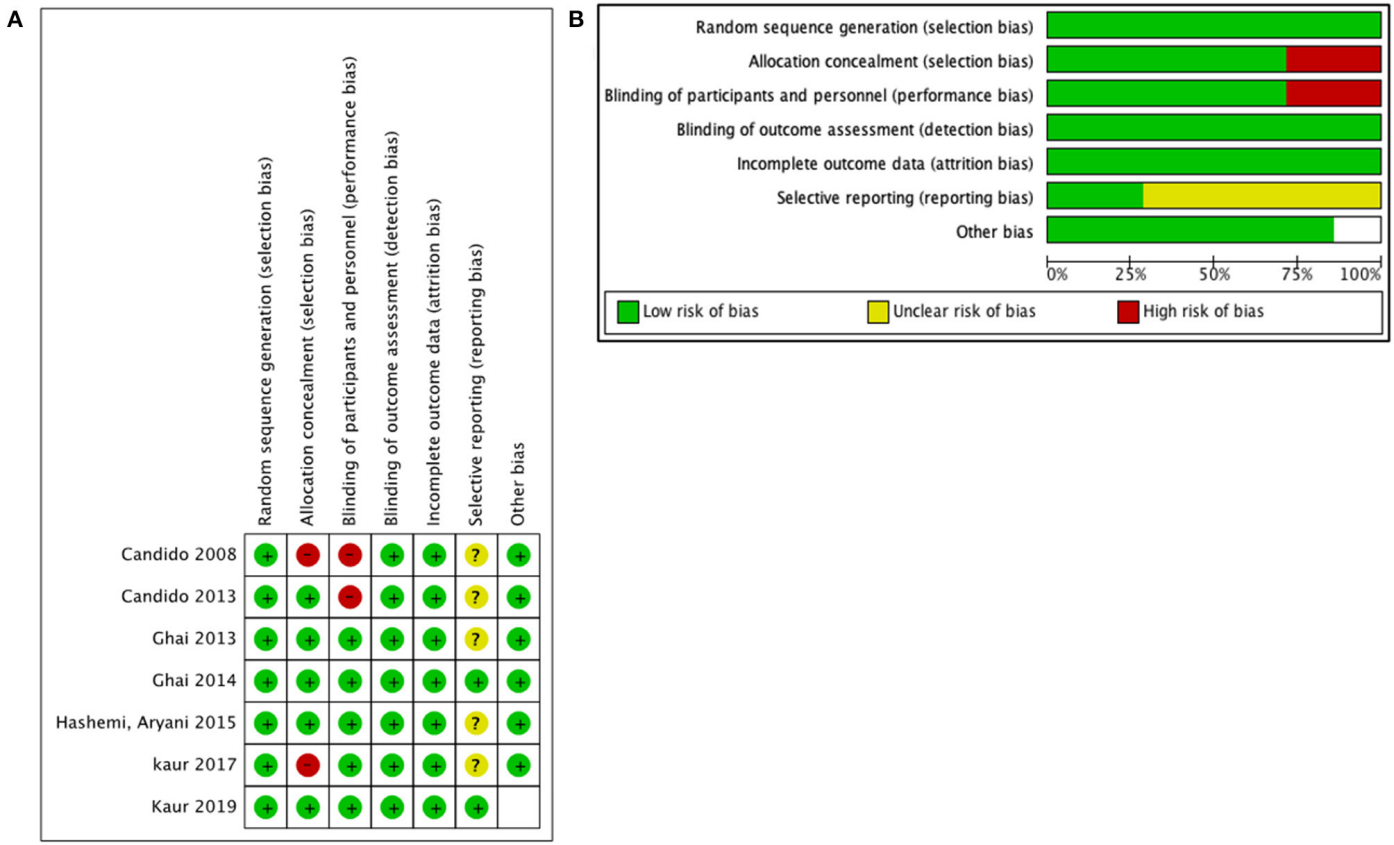
Quality assessment for included studies: **(A)** risk of bias for each prospective randomized study and **(B)** risk of bias graph for all studies.

The risk of bias in the selected studies was measured with the Cochrane Collaboration risk of bias tool assessment (Figures 2A,B). The reporting bias was unclear in the majority of the selected studies. This was secondary to an unavailable protocol to verify the initial interests and outcomes of each study. However, there was a low risk of other biases in the majority of the studies.

### Statistical Analysis

The meta-analysis was performed separately using Review Manager 5.4 software when available data could be combined. The mean and SD with 95% CIs were used to calculate the change in means for the variables related to pain perception and functionality. Data were plotted in forest plots, and treatment effects were evaluated. Heterogeneity was tested by calculation of the *I*-squared (*I*^2^) index and Cochran's Q test; when *I*^2^ > 50% and *P* < 0.1, random effects model was performed. If there were an absence of significant heterogeneity, the Mantel–Haenszel fixed-effects method was performed to pool the estimates. Publication bias was assessed by examination of funnel plots of the estimated effect size on the horizontal axis vs. a measure of study size (SE for the effect size) on the vertical axis. Additionally, the Duval and Tweedie trim-and-fill method was used to impute missing studies, if any, and to recompute an adjusted combined effect.

### Limitations

Few studies have been published comparing specifically the PIL approach with the MIL and TF approaches. The meta-analysis included both RCTs and observational studies, the latter being more prone to biases, specifically selection bias. The same fact could have contributed to the need to perform random effects model analysis due to significant heterogeneity for some of the outcomes; however, no subgroup analysis or meta-regression was performed to identify any specific variable as a potential cause of heterogeneity. Only studies available in the database PubMed were accessed.

## Results

### Study Selection

**Figure 3:**
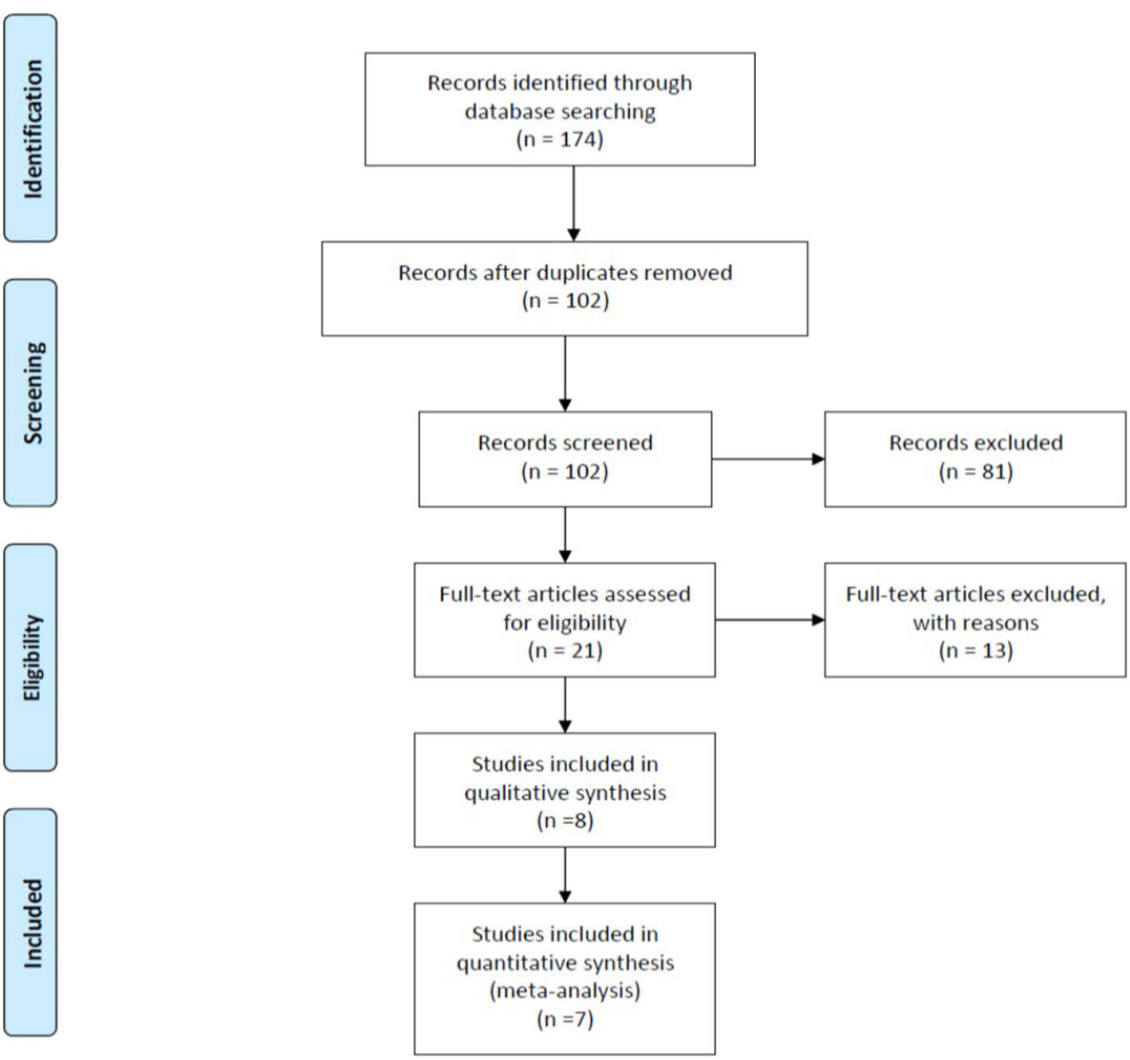
Flow diagram illustrating published literature included. From Moher et al. (32).

We initially identified 174 studies potentially relevant and useful for this review and meta-analysis. After the elimination of duplicates and screening according to that indicated in the “Abstract” section, we eliminated 72 studies that did not meet the inclusion criteria. Finally, eight studies were included in the qualitative analysis, and seven RCTs were included in the statistical analysis. The information was completely reviewed and verified by two authors (S.P and S.C). Figure 3 shows the flow diagram of the study selection process, using the standard recommendations of PRISMA.

### Study Characteristics

The characteristics of each included study are summarized in Table 1. It contains the names of the authors, country, year of publication, type of study design, primary and secondary outcomes, follow-up period, type of approach that was used, number of patients included in each group, and the main findings. All the included studies evaluated the pain change and functionality level change before and after the procedure. The pain was measured with either VAS or NRS, from 0 being no pain to 10 being the highest level of pain. ODI was used as a unique measure of functionality level. The follow-up period ranged from 1 h to 1 year after the procedure. A total of eight studies were finally included for qualitative analysis and seven studies for the meta-analysis; three studies compared the TF approach with the PIL, two studies evaluated the MIL approach compared with PIL, and two studies compared all three approaches.

### Meta-Analysis Results

#### Parasagittal vs. Midline

##### Back-Pain

**Figure 4:**
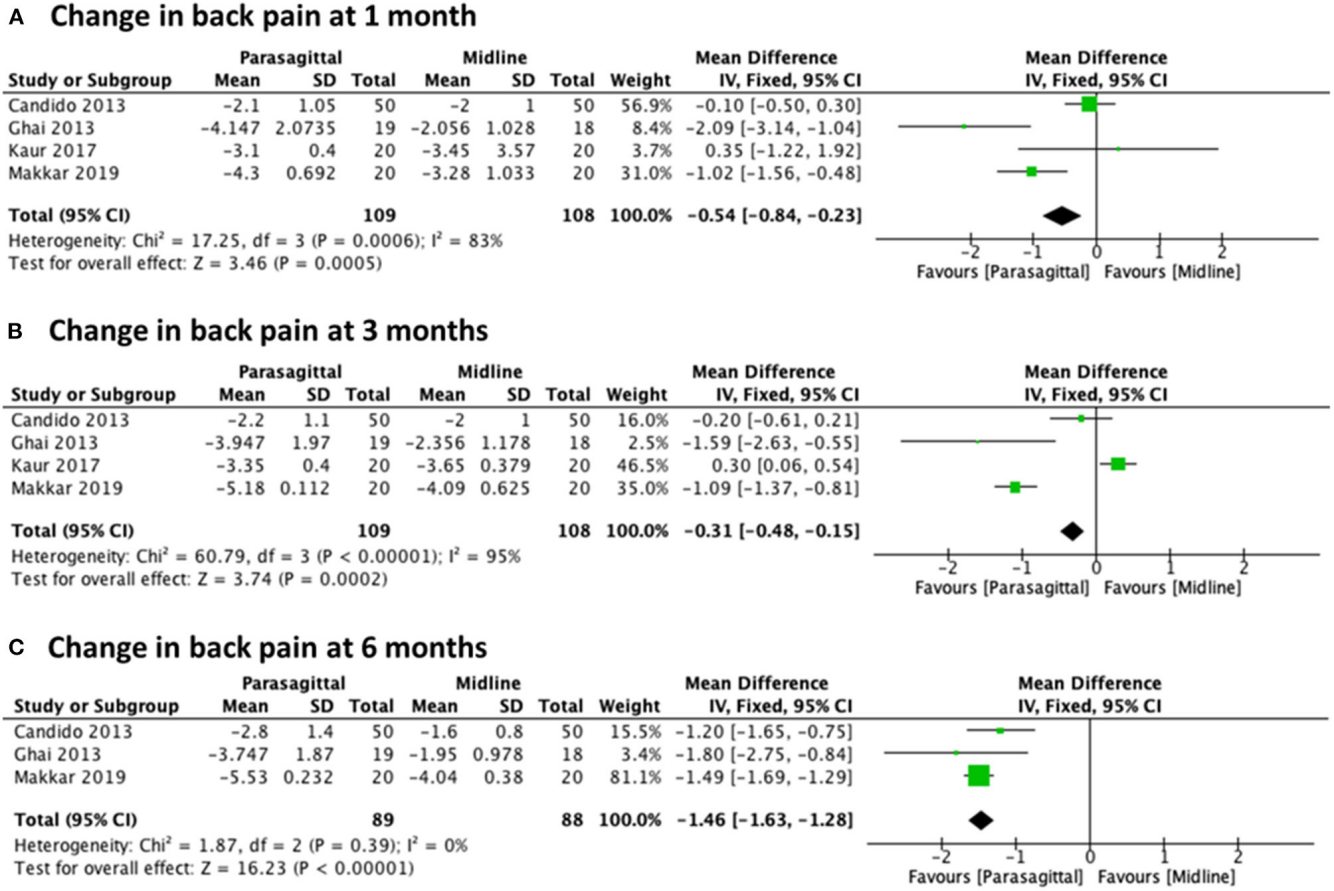
Forest plot comparing the change in back pain between PIL and MIL approaches at **(A)** 1 month, **(B)** 3 months, and **(C)** 6 months.

We grouped and compared the collected data based on the approach used. Change in pain level was compared for the PIL and MIL approaches at 1, 3, and 6 months after the procedure. Of the seven included studies, there were four studies (22, 27, 29, 30) that provided results eligible for analysis of back pain level using NRS, with a total of 217 patients (109 PIL and 108 MIL) for 1 and 3 months, and three trials (22, 27, 29) with a total of 177 patients (89 PIL and 88 MIL) for 6 months. When pooling the effects, the results showed a statistically significant difference in favor of the PIL approach compared with MIL at every follow-up evaluation: [MD −0.54 (−0.84, −0.23), *p* = 0.0005] with high heterogeneity (*I*^2^ = 83%) at 1 month (Figure 4A), [MD −0.31 (−0.48, −0.15), *p* = 0.0002] with high heterogeneity (*I*^2^ = 95%) at 3 months (Figure 4B), and [MD −1.46 (−1.63, −1.28), *p* < 0.00001] with heterogeneity of (*I*^2^ = 0%) at 6 months (Figure 4C).

##### Oswestry-Disability-Index

**Figure 5:**
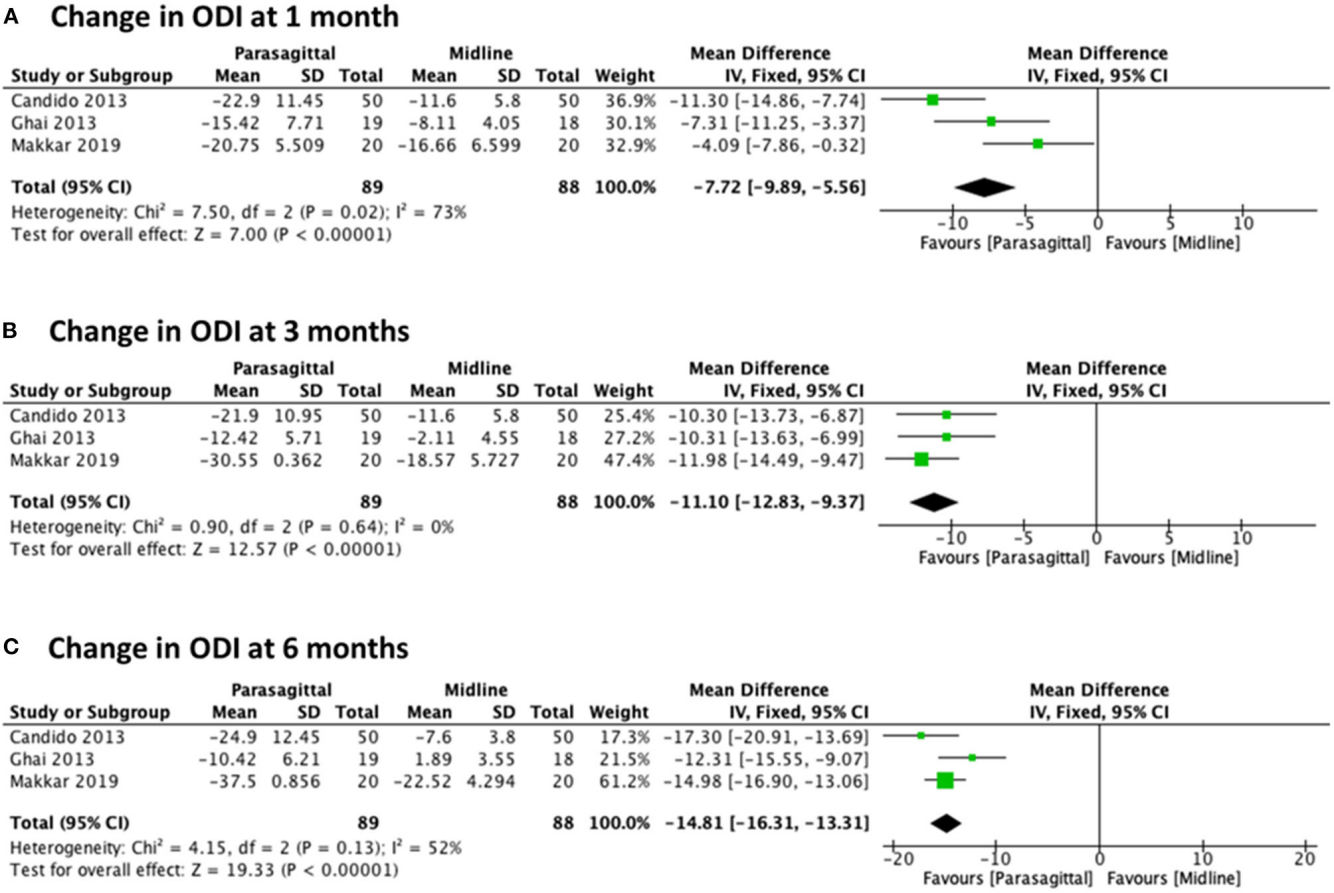
Forest plot comparing the change in ODI between PIL and MIL approaches at **(A)** 1 month, **(B)** 3 months, and **(C)** 6 months.

We also evaluated the change in functional levels at 1, 3, and 6 months after lumbar epidural injection using PIL vs. MIL. For all these three different observed times, three randomized trials were included (22, 27, 29) with a total of 177 patients (89 PIL and 88 MIL). The analysis showed statistical significance in favor of the PIL approach regarding significant functional improvement at all follow-up times evaluated, with [MD −7.72 (−9.89, −5.56), *p* < 0.00001] with high heterogeneity (*I*^2^ = 73%) at 1 month (Figure 5A), [MD −11.10 (−12.83, −9.37), *p* < 0.00001] with heterogeneity of (*I*^2^ = 0%) at 3 months (Figure 5B), and [MD −14.81 (−16.31, −13.31), *p* < 0.00001] with moderate heterogeneity (*I*^2^ = 52%) at 6 months (Figure 5C).

#### Parasagittal vs. Transforaminal

**Figure 6:**
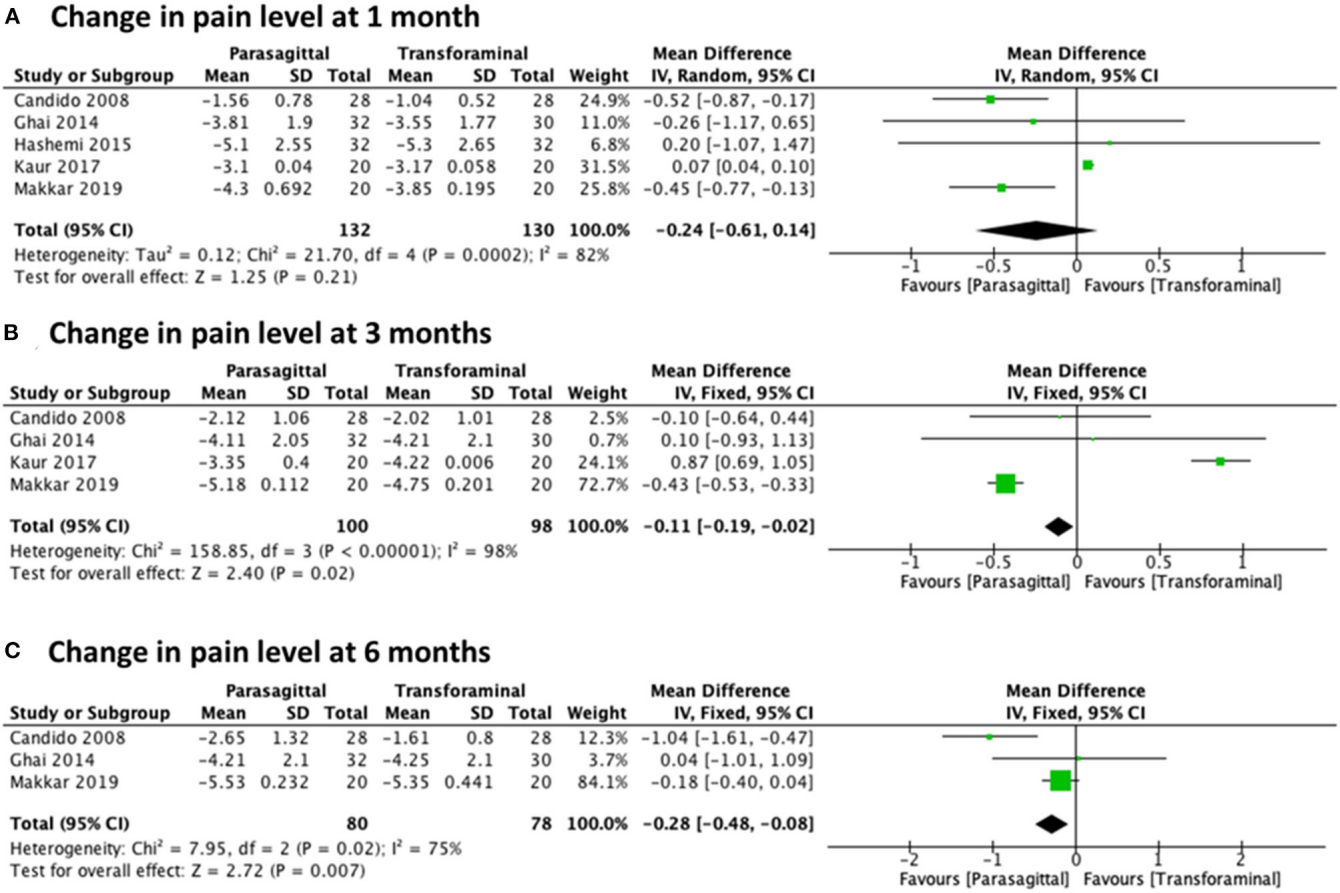
Forest plot comparing the change in pain level between PIL and TF approach at **(A)** 1 month, **(B)** 3 months, and **(C)** 6 months.

The analysis of the change in pain level while comparing the PIL approach and the TF approach included five studies (23, 27–30) for 1 month with a total of 262 patients (132 PIL and 130 TF), four studies (23, 27, 29, 30) for 3 months with a total of 198 patients (100 PIL and 98 TF), and three studies (23, 27, 30) for 6 months with a total of 158 patients (80 PIL and 78 TF). Meta-analysis results demonstrated statistical significance in favor of the PIL approach for pain improvement after 3 and 6 months of follow-up with [MD −0.24 (−0.61, 0.14), *p* = 0.21] with high heterogeneity (*I*^2^ = 82%) at 1 month (Figure 6A), [MD −0.11 (−0.19, −0.02), *p* = 0.02] with high heterogeneity (*I*^2^ = 98%) at 3 months (Figure 6B), and [MD −0.28 (−0.48, −0.08), *p* = 0.007] with high heterogeneity (*I*^2^ = 75%) at 6 months (Figure 6C).

There were not enough studies to pull information from to assess at functional level when comparing PIL vs. TF approaches of lumbar epidural injections.

### Additional Outcomes

Surgical rates, procedure time, contrast spread of epidural injection pattern, and complication rates were extracted. A summary of these results is presented in Table 1. The PIL approach showed benefits in contrast to the other two approaches in terms of less mean fluoroscopy time, less radiation exposure, zero adverse events in all the included studies, no cases of intravascular spread compared with the TF approach, and a higher AES spread of PIL (100%) compared with TF (75%).

## Discussion

The main findings of this systematic review and meta-analysis suggest significant statistical data in favor of the PIL approach. The results demonstrate a larger decrease in low back pain score and an increase in the patient functionality when compared with both MIL and TF approaches. Although there are few studies in the literature and contradictory data, our quantitative analysis advocates the modified PIL approach as an alternative option. When performed by an expert pain physician, it is a promising procedure, reducing procedure time and incidence of adverse events such as exposure to radiation and intravascular injection.

Nowadays, MIL and TF are the two approved standard procedures for low back pain secondary to radiculopathy. However, they are associated with multiple complications, from intravascular injection to spinal cord injury, and even permanent paralysis (33). Lately, the PIL approach has been one of the areas of focus in an effort to find a more reliable, safe, and suitable approach.

The first recorded use of the PIL approach, also known as the paramedian approach, was described by Bonica in 1956 (34). It is a modified interlaminar epidural injection performed at the most lateral part of the interlaminar space rather than the midline space. The needle is inserted at 1–1.15 cm lateral of the midline and follows toward the targeted lesion that lies away from the spinal vessel plexus (Figure 1). Within its benefits, PIL allows superior medication delivery compared to other approaches. This was demonstrated in a study that compared the AES spread of iodine when using the PIL vs. TF approach. After blinded radiologists evaluated the distribution pattern, the results showed an anterior distribution of 100% (29 out of 29) with the PIL approach, compared to 75% (21/28) with the TF approach (23). These findings could potentially suggest a greater reduction of pro-inflammatory cytokines, due to a more direct spread of the drug, and thus a better outcome to the therapy.

Despite the multiple benefits of the TF technique, it also showed a higher incidence of severe complications. Intravascular injection is the most commonly reported technique, with an incidence of 11.2% with TF vs. 1.9% with the IL approach (35, 36). Also, cases of paraplegia due to spinal cord infarction have been shown to be associated with particulate (“insoluble”) use of steroid, causing the occlusion of the segmental artery or vertebral artery (33, 35, 37, 38).

To reduce intravascular injection, without decreasing the effectiveness of the therapy, PIL is being considered as an alternative technique, given its ability to avoid the spinal vessel plexus (23, 27, 28). In this approach, after the needle passes through the ligamentum flavum, it should be directed away from the plexus, in contrast to the TF approach, where the needle advances directly into the blood vessel-rich area. On the other hand, in the MIL approach, the needle bypasses this vessel-rich area; however, this technique does not deliver a reliably high dose of the drug to the target zone. Ghai et al. also evaluated cases of intravascular spread of PIL compared with the TF approach, and no cases were found in the PIL group, while three cases were identified in the TF group (27). Thus, the modified PIL approach seems to be a better option to avoid possible adverse events with this type of therapy.

Multiple studies have evaluated the changes in pain and functional levels comparing different epidural injection approaches (16, 22, 23, 26, 27, 29, 39, 40). A randomized study carried out by Ackerman and Ahmad demonstrated better pain relief in patients receiving ESI through the TF approach vs. both the MIL and caudal approach, possibly due to a higher concentration of drug delivery directly to the target zone (41). Similarly, Schaufele et al. compared MIL and TF approaches and reported the TF approach to be superior in pain improvement and to have a less long-term need for surgical intervention (40). Candido et al. evaluated the concordant pressure paresthesia phenomenon, which correlates with pain relief in patients with unilateral radicular pain, between the MIL and PIL approaches. Although both techniques showed statistically and clinically significant pain relief, the PIL technique had a slightly better control of pain and functionality improvement. Additionally, patients who received PIL had a higher concordant moderate-to-severe pressure paresthesia, which was associated with a higher and long lasting pain improvement (26). Ghai et al. found a higher incidence of effective pain improvement at 6 months in the PIL group (68.4%), compared with the MIL group (16.7%) (22). These findings are comparable with our meta-analysis results.

After our data were pooled and the quantitative analysis was performed, the results demonstrate a statistically significant difference regarding the change in pain level, favoring the PIL approach in short term (1 month) and long term (6 months) compared with MIL, and the MIL in short term (3 months) and long term (6 months) compared with TF. This new evidence in favor of the PIL approach could be explained due to a higher sample size, which increases the weight/power of the results, and at the same time, the study design that allows us to decrease possible confounders in previous studies, due to the heterogeneity present on those analyses.

In addition, the PIL approach is a cost-effective epidural injection that requires a less demanding procedural technique, less procedural time, decreased radiation exposure, low complication rates, better consistency for reaching the AES, and a strong safety profile (42). The use of steroid is also a controversial topic. Systematic reviews evaluated the response of the addition of corticosteroids to local anesthetics compared with local anesthetics alone in epidural injections for chronic low back pain. The results showed an increase in pain relief when using steroids in all types of approaches (IL, TF, and caudal epidural) (2, 43, 44). Some evidence discourages the use of steroids due to catastrophic vascular complications, usually associated with high rates of intravascular injections. As PIL reduced the risk of intravascular injection, this approach could be a safer technique when using steroids in epidural injections (42).

There are some limitations to our study. First, we identified heterogeneity in the type and concentration of steroid drugs used in each study, which could potentially affect the outcomes of each technique. Second, PIL is a newly modified and underutilized approach by pain physicians; therefore, few studies are published, which explain our small sample size. Also, although initially more studies were identified, the extracted data were incomplete, and even after reaching out to the authors, the majority did not meet the inclusion criteria, further reducing the sample size. The risk of bias was measured with the Cochrane Collaboration risk-of-bias tool assessment, showing a low risk of bias.

The results of this meta-analysis suggest that the PIL approach could be a suitable alternative to TF and MIL techniques for ELIs in patients with chronic radicular low back pain secondary to disc herniation or nerve root inflammation. The results demonstrate a statistically significant pain relief of up to 6-months after procedure with the PIL approach, beginning as early as 1 month compared to MIL, and as early as 3 months compared to TF. In addition, functionality level was also better in the PIL group compared with MIL. Therefore, we strongly encourage the modified PIL technique to be performed by an experienced pain physician, as an efficient, safe, and cost-effective option, for patients with chronic low back pain when epidural injections are indicated.

## Data Availability Statement

The raw data supporting the conclusions of this article will be made available by the authors, without undue reservation.

## Author Contributions

NK helped with the development of the protocol and search strategy, selection of studies, interpretation of analyses, drafting the article, and final review. SP helped with the development of the protocol and search strategy, selection of studies, risk of bias assessment, data extraction and analyses, interpretation of analyses, drafting the article, and final review. SC helped with the selection of studies, risk of bias assessment, data extraction and analyses, interpretation of analyses, and final review. AH helped with the development of the protocol and search strategies, making figures, drafting the article, and final review. KC helped with the development of the protocol, selection of studies, and final review. All authors contributed to the article and approved the submitted version.

## Conflict of Interest

The authors declare that the research was conducted in the absence of any commercial or financial relationships that could be construed as a potential conflict of interest.
